# Evaluation of Total Isoflavones in Chickpea (*Cicer arietinum* L.) Sprouts Germinated under Precursors (*p*-Coumaric Acid and L-Phenylalanine) Supplementation

**DOI:** 10.3390/plants12152823

**Published:** 2023-07-31

**Authors:** Jaya Arora, Bhanupriya Kanthaliya, Abhishek Joshi, Mukesh Meena, Supriya Meena, Manzer H. Siddiqui, Saud Alamri, Hari Prasad Devkota

**Affiliations:** 1 Laboratory of Biomolecular Technology, Department of Botany, Mohanlal Sukhadia University, Udaipur 313001, Rajasthan, Indiaabhijoshi2015@gmail.com (A.J.);; 2 Laboratory of Phytopathology and Microbial Biotechnology, Department of Botany, Mohanlal Sukhadia University, Udaipur 313001, Rajasthan, India; 3 Department of Botany and Microbiology, College of Science, King Saud University, Riyadh 11451, Saudi Arabia; mhsiddiqui@ksu.edu.sa (M.H.S.); saualamri@ksu.edu.sa (S.A.); 4 Graduate School of Pharmaceutical Sciences, Kumamoto University, Oe-Honmachi, Chuo-ku, Kumamoto 862-0973, Japan; devkotah@kumamoto-u.ac.jp

**Keywords:** chickpea, isoflavone, antioxidant, morphological growth, nutrients, functional food

## Abstract

*Cicer arietinum* L. (Bengal gram, chickpea) is one of the major pulse crops and an important part of traditional diets in Asia, Africa, and South America. The present study was conducted to determine the changes in total isoflavones during sprouting (0, 3, and 7 days) along with the effect of two precursor supplementations, *p*-coumaric acid (*p*-CA) and L-phenylalanine (Phe), in *C. arietinum*. It was observed that increasing sprouting time up to the seventh day resulted in ≈1282 mg 100 g^−1^ isoflavones, which is approximately eight times higher than chickpea seeds. The supplementation of Phe did not affect the total length of sprouts, whereas the supplementation of *p*-CA resulted in stunted sprouts. On the third day of supplementation with *p*-CA (250 mg L^−1^), the increase in the total phenolic content (TPC) (80%), daidzein (152%), and genistin (158%) contents were observed, and further extending the supplementation reduced the growth of sprouts. On the seventh day of supplementation with Phe (500 mg L^−1^), the increase in TPC by 43% and genistin content by 74% was observed compared with non-treated sprouts; however, the total isoflavones content was found to be 1212 mg 100 g^−1^. The increased TPC was positively correlated with the 2,2-diphenyl-1-picrylhydrazyl (DPPH) radical scavenging activity (r = 0.787) and ferric-reducing antioxidant potential (FRAP) (r = 0.676) activity. This study suggests that chickpea sprouts enriched in TPC and antioxidants can be produced by the appropriate quantity of precursor supplementation on a particular day. The results indicated major changes in the phytochemical content, especially daidzein and genistin. It was also concluded that the consumption of 100 g of seventh-day sprouts provided eight times higher amounts of isoflavones in comparison to chickpea seeds.

## 1. Introduction

Pulses are grown worldwide and are a rich source of protein, dietary fibers, minerals, and bioactive substances such as polyphenols, phytosterols, saponins, etc. that exert various pharmacological effects [1,2]. *Cicer arietinum* L. (Bengal gram, chickpea) is one of the ancient pulse crops and is extensively consumed due to its rich source of protein. It is rich in polyphenolic compounds including flavonols, flavone glycosides, and oligomeric and polymeric proanthocyanidins [3,4]. Isoflavones are a class of phytochemicals with structural similarities to mammalian estrogens known as phytoestrogens. They possess both estrogenic and anti-estrogenic properties, and research has shown potential benefits in reducing the risk of chronic diseases, such as cardiovascular diseases, osteoporosis, and certain types of cancer [5,6,7]. The safe daily intake of isoflavones has not yet been established for humans, but the Food and Drug Administration (FDA) recommends an intake of 40–120 mg per day [8].

Sprouting is a process that involves seed germination and leads to sprout development. Sprouting increases the nutritional and bioactive content of seeds while reducing antinutritional compounds, such as trypsin inhibitors, lectins, phytates, and tannins [9]. Several biochemical and physiological changes occur during sprouting, resulting in an increase in the content of various beneficial compounds, including vitamins, minerals, polyphenols, and other phytochemicals [10]. In addition, sprouting activates several hydrolytic and proteolytic enzymes, including amylases, proteases, and peptidases, which break down macromolecules into simpler forms that are easily digested and absorbed by the human body [11]. Legumin and vicilin, major legume seed storage globulins, were reported to be more digestible because of their altered structural conformation after germination [12]. It has also been reported that germinating legume sprouts show marked increases in the total amount of free amino acids including asparagine, glutamine, and alanine, but their extent of improvement in digestibility is species-specific [13].

Phenylpropanoids are plant-specific metabolites, and their biosynthetic pathways are extremely diverse. They are precursors of a variety of metabolites that regulate plant growth, metabolism, and interactions with environmental stimuli, such as biotic and abiotic stresses [14,15,16,17,18,19]. They have also been utilized in food supplements, pharmaceuticals, and cosmetics, and have numerous advantages for human well-being [20,21]. Chickpea seeds and sprouts contain diverse phenylpropanoids that contribute to their immense antioxidant, estrogenic, and antiproliferative activities [22].

In recent years, researchers have explored strategies to further enhance the bioactive content of legumes by manipulating the germination conditions. One such approach involves the use of exogenous precursors or elicitor compounds to stimulate the synthesis of specific bioactive compounds [23,24,25,26,27]. Precursors are compounds that can be metabolized within a plant system to produce targeted bioactive compounds [28]. This approach has been successfully utilized to increase the levels of various health beneficial compounds in legumes, glucosinolates in broccoli sprouts [29], polyphenols in mung beans [30,31,32,33], and antioxidants in soybeans, black beans, and lentils sprouts [34,35,36]. In a recent study, Yin et al. [37] suggested that melatonin supplementation significantly increased the levels of flavonoids and isoflavones (daidzein, genistein, daidzin, glycitin, and genistin) in 4-day-old germinated soybeans.

*p*-Coumaric acid (*p*-CA), an upstream metabolite of the phenylpropanoid pathway, is a common precursor for the biosynthesis of numerous derivatives, including flavonoids, stilbenes, and anthocyanins [38,39]. Aromatic amino acids, such as L-phenylalanine (Phe), tyrosine, and tryptophan, are also functional precursors for a variety of secondary metabolites required by both plants and humans [40,41]. Therefore, this study evaluated the effects of *p*-CA and Phe supplementation on morphological and phytochemical attributes (total phenolic content, total isoflavone content, and antioxidant activity) in chickpea sprouts during germination. This study also aimed to provide insights into the optimization of sprouting conditions to maximize the nutritional value of chickpea sprouts.

## 2. Result and Discussion

### 2.1. Effect of Precursors on the Morphological Parameters of C. arietinum Sprouts

**Figure 1:**
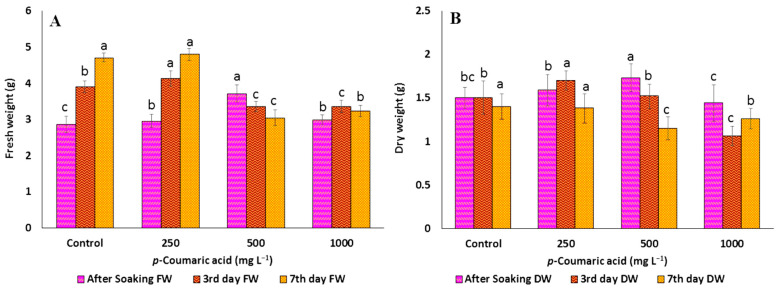
(**A**) Fresh weight and (**B**) dry weight of *C. arietinum* sprouts of the control and different concentrations of *p*-coumaric acid. Data represented mean ± SD calculated from at least three replicates of each sample; means with different letters are significantly different at *p* ≤ 0.05 according to Duncan’s multiple range tests (DMRT).

**Figure 2:**
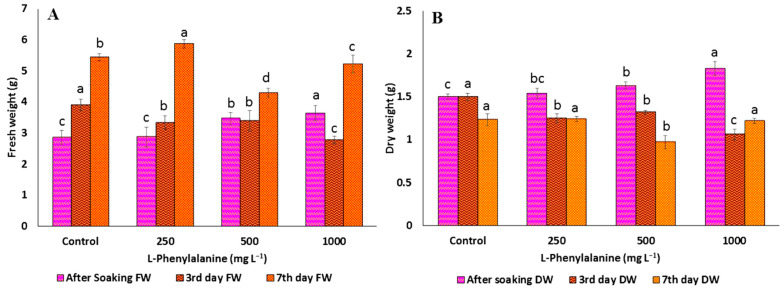
(**A**) Fresh weight and (**B**) dry weight of *C. arietinum* sprouts of the control and different concentrations of L-phenylalanine. Data represented mean ± SD calculated from at least three replicates of each sample; means with different letters are significantly different at *p* ≤ 0.05 according to Duncan’s multiple range tests (DMRT).

**Figure 3:**
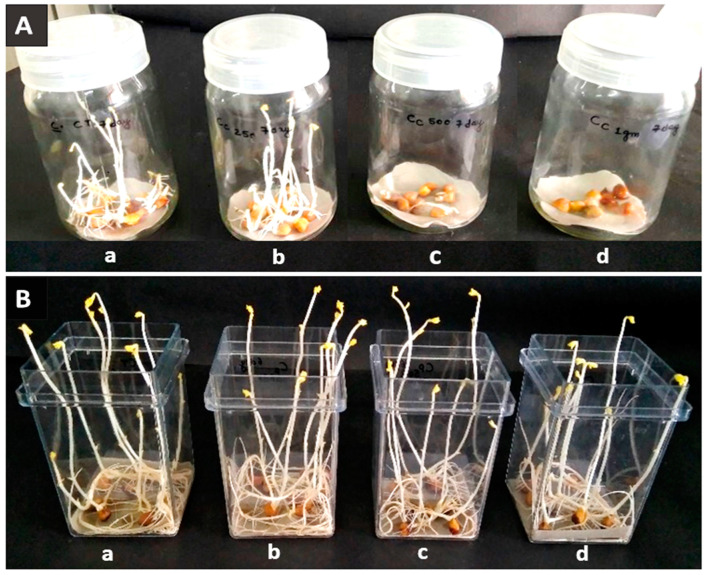
*Cicer arietinum* sprout at the 7th day. (**A**) *p*-coumaric acid, (**B**) L-phenylalanine, (**a**) control, (**b**) 250 mg L^−1^, (**c**) 500 mg L^−1^, (**d**) 1000 mg L^−1^, respectively.

The allelochemicals produced by plants are the secondary metabolites that can positively or negatively influence the growth and development of many organisms [42]. It has been widely established that *p*-CA has allelopathic effects on morphological and physiological processes of root growth [43,44]. In the present study, chickpea germination percentage, as well as length of hypocotyl and radical, decreased significantly with increasing concentrations of *p*-CA, reaching a complete reduction at higher concentrations (Table 1 and Figure 1A). The FW and DW were significantly increased (5.7% and 13%) up to 250 mg L^−1^, but after that concentration, FW (Figure 2A) and DW (Figure 2B) were significantly decreased (14 and 29.3%, respectively). Similarly, reduction in total germination, i.e., hypocotyls and radical growth, was seen at 250 mg L^−1^ concentration up to the third day of soaking, i.e., 80% (0.25 and 1.53 cm, respectively). On the other hand, the trends of growth were similar at the different concentrations of Phe. Phe was observed to improve growth parameters, such as total germination, FW, DW (increase by 3, 5, and 7% at 250, 500, and 1000 mg L^−1^, respectively), length of hypocotyls (0.87 cm at 1000 mg L^−1^, 2.72 cm at 250 mg L^−1^), and radical (3.10 cm at 250 mg L^−1^, 12.72 cm at 250 mg L^−1^) (Figure 1B). Compared to the control, most of the improvement was observed up to 3 days after soaking, but thereafter, no noticeable improvement was observed (Table 2 and Figure 3A,B). Previous studies reported that soaking seeds in the presence of *p*-CA inhibited water uptake, electrolyte retention capacity, and O_2_ consumption [45,46,47]. Insufficient and/or late seed rehydration caused by *p*-CA could have delayed membrane stabilization or decreased respiratory oxygen consumption, both of which are conducive to an overproduction of reactive oxygen species [48]. Being unbalanced by a sufficient increase in antioxidant defense systems, the resulting oxidative stress might have eventually interfered with the germination program. In the case of Phe, amino acids as biostimulants (substances that promote plant growth, improve nutrient availability, and enhance plant quality) are not only getting popular for mitigating injuries caused by abiotic stresses [49] but also serve as hormone precursors [50], signaling factors of different physiological progressions, such as glutamate receptors (GRLs) [51], and regulators of nitrogen uptake [52,53] and root development [54].

### 2.2. Effect of Precursors on Total Phenolic Contents of C. arietinum Sprouts

It was observed that in the control conditions when no supplementation of *p*-CA was provided, the TPC content in chickpea sprouts showed an increase from zero day (4.3 mg GAE g^−1^ DW) to the seventh day of sprouting (18.5 mg GAE g^−1^ DW). Soaking for 24 h in *p*-CA, the maximum TPC content (9.3 g GAE g^−1^ DW) was obtained at 500 mg L^−1^. Treatment with 250 mg L^−1^ *p*-CA resulted in 17.6 mg GAE g^−1^ DW TPC on the seventh day. Higher concentrations of *p*-CA (500 mg L^−1^, 1000 mg L^−1^) resulted in a non-significant change in TPC (Table 3). Phe (250 mg L^−1^) was found to be very effective at the initial 1-day soaking of chickpea seeds by increasing TPC (8.1 mg GAE g^−1^ DW) twice compared to non-treated seeds, which further tripled (14.9 mg GAE g^−1^ DW) at the third day. An increase in TPC (24.3 mg GAE g^−1^ DW) was observed up to the seventh day of sprouting. However, a higher TPC (32.3 mg GAE g^−1^ DW) content was obtained with 500 mg L^−1^ Phe, which was further reduced at higher Phe concentrations (Table 4).

Phenolic compounds can be found in the free form or conjugated with other molecules; therefore, their bioavailability and the pathways via which they are metabolized change according to their chemical structure. According to the literature, free *p*-CA and *p*-CA conjugates appear to be associated with different absorption rates and metabolic pathways [55]. These result in a reduction in the activity of some key enzymes required for metabolic continuation and inhibit the transcription of molecular chaperons implicated in secretory pathways. *p*-CA causes late seed rehydration, which results in delayed membrane stabilization or reduced respiratory oxygen consumption, leading to the overproduction of reactive oxygen species. This did not significantly enhance phenolic content and antioxidant properties [56]. Precursor supplementation, such as Phe amino acid, has been successfully used to enhance the production of secondary metabolites in plant cell cultures [57].

### 2.3. Effect of Precursors on Antioxidant Activities of C. arietinum Sprouts

Phenolic compounds are well known for their antioxidant properties; they scavenge reactive oxygen species (ROS) and regulate endogenous antioxidant enzymes, thereby preventing oxidative damage to biomolecules [58,59,60]. The methanolic extracts were able to reduce stable radicals, indicating that the extracts possessed hydrogen-donating capabilities and acted as antioxidants. Slightly enhanced ferric-reducing antioxidant potential was observed at 1000 mg L^−1^ concentration of *p*-CA (7.34 mM Fe^+2^ g^−1^ DW), while DPPH activity was enhanced at 1000 mg L^−1^ concentration (52.45% inhibition). This might be because *p*-CA leads to a reduction in peroxidase enzymatic activity, which activates superoxide dismutase. Subsequently, *p*-CA postponed the activity of the peroxidases enzyme and activated superoxide dismutase, thus reducing the activities of some marker enzymes for metabolic continuation and inhibiting the transcription of molecular chaperons implicated in secretory pathways [61]. Foliar and seed applications of amino acids affect the antioxidant metabolism of soybean crops [62]. The results showed that phenolic content was significantly increased in the DPPH and FRAP assays compared to the control (*p* < 0.05). Enhancement was observed for up to the third day for all the concentrations of Phe, and later on (up to the seventh day), TPC and DPPH decreased at 1000 mg L^−1^ concentration. However, a reduction in the FRAP activity was observed at all concentrations. Phenylalanine ammonia-lyase (PAL) activity was induced by Phe supplementation; hence, increased phenolic contents and antioxidant properties were observed [63,64]. Most importantly, in *C. arietinum* sprouts, total phenolic content is strongly and positively correlated with PAL activity [65].

### 2.4. Correlation between Assays

**Figure 4:**
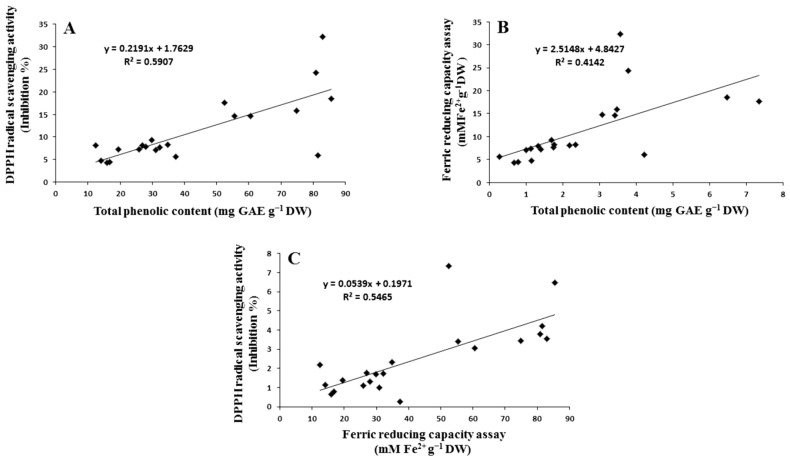
Correlation curve between (**A**) antioxidant activity of TPC and DPPH method, (**B**) antioxidant activity of TPC and FRAP assay method, and (**C**) FRAP and DPPH assay.

The calculated coefficient of correlation (r) between the two antioxidant tests and TPC in the treated sprouts is illustrated in Figure 4, where a significant correlation was observed between TPC and DPPH activity (r = 0.676, Figure 4A), TPC and FRAP activity (r = 0.787, Figure 4B), and FRAP and DPPH activity (r = 0.758, Figure 4C) in sprouts. This indicates that phenolic compounds could contribute to the scavenging activity and reduce the power of treated sprouts; therefore, the presence of phenolic compounds in plant extracts significantly contributes to their antioxidant potential. The antioxidant properties of phenolic compounds are associated with their structures. Indeed, phenolics are composed of one (or more) aromatic rings bearing one or more hydroxyl groups and are therefore potentially able to quench free radicals by forming resonance-stabilized phenoxyl radicals. Sulaiman et al. [66] also found a positive correlation between total phenolic and mineral contents with the antioxidant activity of eight Malaysian bananas (*Musa* sp.). A similar result was obtained by Ismail [67] in *Syzygium polyanthum* (serai kayu) leaf fractions. In the case of TPC, DPPH, and FRAP, Phe was observed to be better than *p*-CA, with a level of significance of *p* ≤ 0.05. TPC was enhanced by 7.1 mg (GAE) g^−1^ DW (r = 0.989) and 8.2 mg (GAE) g^−1^ DW (r = 0.964) immediately after soaking and on the third day of soaking at higher concentrations of *p*-CA. The DPPH activity was enhanced at 1000 mg L^−1^ concentration just after soaking (31.02% inhibition), whereas Phe was observed to be better than *p*-CA and significantly enhanced the phenolic content and antioxidant properties. Maximum increment with a significance level of *p* < 0.05 was observed on the seventh day of soaking at 500 mg L^−1^ concentration for TPC (32.3 mg GAE g^−1^ DW; r 0.359) and on the third day of soaking for DPPH (r 0.886) and FRAP (3.47 mM Fe^+2^ g^−1^ DW; r 0.997). An enhanced level of TPC and antioxidant effect is thought to be an induction of PAL activities after supplementation with Phe [63]. Thus, the correlation also clearly suggested that Phe is better than *p*-CA in improving the biochemical and morphological features in the present study.

### 2.5. High-Performance Liquid Chromatography Analysis of Phenolic Content in Control and Precursor-Treated C. arietinum Sprouts

**Figure 5:**
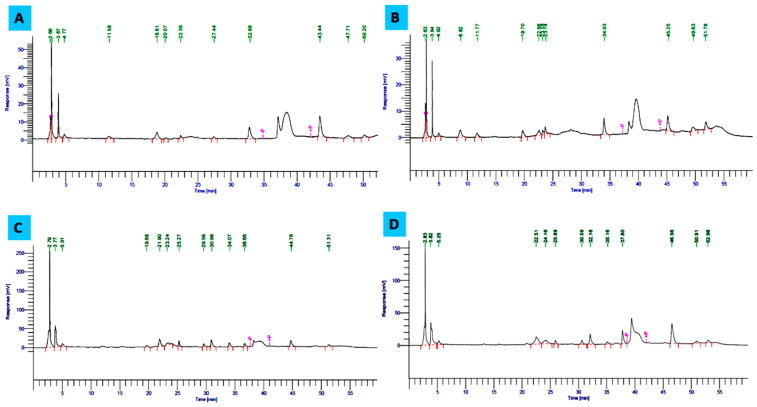
HPLC chromatogram showing the manifestation of isoflavonoid contents in *C. arietinum* sprout. (**A**) third-day control, (**B**) third-day treated with *p*-coumaric acid (250 mg L^−1^), (**C**) seventh-day control, (**D**) seventh-day treated with L-phenylalanine (250 mg L^−1^).

HPLC is one of the most reliable methods to determine the changes in phenolic content in the presence of elicitor compounds or under different growth conditions [23,68]. The HPLC chromatogram (Figure 5A–D) showed an increase in the concentration of some phenolic compounds in the presence of both *p*-CA and Phe supplementations. Multiple peaks in the chromatogram suggest the presence of different conversion products of the isoflavone metabolic pathway. According to Kanthaliya et al. [69] metabolic activity is affected by environmental conditions, which modulate the quality and quantity of isoflavones. Daidzein and genistein are known to be the most common dietary *C. arietinum* isoflavones, and the quantity of both metabolites increased in the presence of precursors under study [68]. Conversion of daidzein to genistein involves the formation of two important metabolites, equol, and 5-hydroxy-equol, which might correspond to the unidentified peaks in the chromatogram. In the presence of *p*-CA, daidzin and genistin are known to be increased by 0.730 and 1.057 mg g^−1^, respectively, and the conversion is slightly lower than that of Phe. At both 250 mg L^−1^ as well as 1000 mg L^−1^ concentrations of Phe, the main increased lead enhancement of metabolic products of isoflavone metabolic pathways were daidzin, daidzein, and genistein. Chromatogram peaks on the seventh day of incubation at 250 mg L^−1^ of Phe depicted an increase in daidzin and genistein by 6.33 and 4.171 mg g^−1^ of peaks value, respectively. Daidzein and genistin were observed unexpectedly high at 500 mg L^−1^ concentration of the same precursor, i.e., 0.534 and 6.842 mg g^−1^, respectively (Table 5). However, the total isoflavone content was observed to be less than that of the control, which might be due to the complexity of the phenylpropanoid pathway leading to the production of undetectable isoflavone metabolites showing differential elution behavior. Isoflavones are usually synthesized as part of the phenylpropanoid pathway. The phenylpropanoid pathway has multiple branches common to legumes, which provide numerous compounds including lignins, anthocyanins, and certain classes of phytoalexins that have roles in normal development, as well as serving as protectants against many environmental stresses [70,71]. Arrays of genes encoding enzymes of the pathway are developmentally and tissue-specifically regulated and induced in the presence of precursors or elicitors [70]. These genes include PAL, chalcone synthase (*c2*), chalcone isomerase (*chi*), flavanone 3-hydroxylase (*f3h*), flavanone/dihydroflavonol reductase (*a1*), proanthocyanidin synthase (*a2*), UDP-Glc: flavonoid 3-*O*-glucosyltransferase (*bz1*), and a glutathione*S*-transferase (*bz2*) [72]. Thus, the peaks suggested that precursors such as Phe lead to enhanced induction of the metabolic conversion of isoflavones and stimulate increased synthesis of therapeutic compounds such as daidzin, daidzein, and genistein, which are known for prevention of hormone-dependent and age-related diseases, including cancer, osteoporosis, menopausal symptoms, and cardiovascular diseases [73,74].

## 3. Materials and Methods

### 3.1. Plant Genotype

Chickpea variety C.CSCD-884 (moderate resistant to wilt, dry root rot) was procured from Agricultural Research Station, Durgapura, Jaipur, Rajasthan, India. This chickpea variety seeds matrix is comprised of protein (≈23%), carbohydrates (≈59%), moisture (≈8%), fat (≈11%), and micronutrients (≈1%). This chickpea variety provides 5.9 mg of iron (Fe), 4.5 mg of zinc (Zn), 52.5 mg of selenium (Se), 145 mg of magnesium (Mg), 190 mg of calcium (Ca), 0.9 mg of copper (Cu), 1024 mg of potassium (K), and 355 mg of phosphorus (P) in a 100 g serving.

### 3.2. Reagents

Phenylalanine, *p*-coumaric acid, gallic acid, 2,2-diphenyl-1-picrylhydrazyl (DPPH), 2,4,6-tris[2-pyridyl]-*s*-triazine (TPTZ; MW: 312.33), ferric chloride, methanol, and all other reagents and chemicals used were of analytical grade. Solvents used for HPLC analysis were of HPLC grade. All reagents and chemicals are procured from Himedia Pvt. Ltd., Mumbai, India.

### 3.3. Sprouting Procedure

*Cicer arietinum* seeds were placed on a 5 cm thick cotton bed in a magenta box (PLANTON, Tarson, Kolkata, India) with 4 mL double-distilled water and investigated for growth with control (without supplementation) and 24 h soaking supplementation of *p*-CA (250 mg L^−1^, 500 mg L^−1^, and 1000 mg L^−1^) and Phe (250 mg L^−1^, 500 mg L^−1^, and 1000 mg L^−1^). The magenta boxes were placed in a BOD incubator at 27 ± 0.1 °C temperature. The seeds sprouted up to the 7th day from incubation day, with that day being zero day.

### 3.4. Study of Morphological Parameters

Using different germination growth parameters such as total germination (%), fresh weight (g), dry weight (g), length of hypocotyl, and radicle (cm) after soaking, 3rd- and 7th-day germinated sprouts were measured using the measuring scale and weighing machine (Model BT 323 S, Sartorius, Goettingen, Germany).

### 3.5. Preparation of the Extract

After soaking, the 3rd- and 7th-day sprouts were dried at 60 °C in the oven for 12 h, and after that, the sprouts were ground in a pastel-motor. The extract was prepared (cold extraction method) by dissolving 250 mg of dried powder of sprouts in 5 mL of 70% methanol and shaking on the test tube rotator (Model Abdos waves) for 12 h at 70 rpm speed at room temperature (24–26 °C). After that, sonication for 10 min was performed using a sonicator (Sonar), and the extract was centrifuged for 10 min on 3000× *g*. The supernatant was collected, and the extracts were stored at −20 °C before biochemical analysis.

### 3.6. Determination of Total Phenolic Content

Phenolic compounds extracts of the sprouts were analyzed using the Foline–Ciocalteu reagent, followed by the method of Farkas and Kiraly [75]. TPC was expressed as mg gallic acid equivalents (GAE) g^−1^ DW using the standard curve with gallic acid at 650 nm.

### 3.7. Determination of Antioxidant Activities

#### 3.7.1. DPPH Radical Scavenging Activity

DPPH radical scavenging activity was determined according to the method described by Hatano et al. [76] with slight modification. The reaction mixture (total volume 3 mL), consisting of 0.1 mL of extract, 0.5 mL of 0.5 M acetic acid buffer solution at pH 5.5, 1 mL of 0.2 mM DPPH in ethanol, and 1.4 mL of 50% (*v*/*v*) ethanol aqueous solution, was shaken vigorously with various samples. After incubation at room temperature for 30 min, the remaining DPPH was determined by absorbance at 517 nm, and the radical scavenging activity of each sample was expressed using the ratio of the absorption decrease in DPPH radical scavenging activity (%) to that of the control DPPH solution (100%) in the absence of the sample. The radical scavenging activity was calculated as (%) = 100 (A − B)/A, where A and B are absorptions of the control (50% ethanol without sample and DPPH) and the sample (sample reaction mixture with DPPH), respectively.

#### 3.7.2. Ferric-Reducing Antioxidant Potential (FRAP) Assay

The ferric-reducing power of the plant extracts was determined using the method of Benzie and Strain [77] with slight modifications. The reaction mixture, containing 100 µL of sample solutions, 300 µL of deionized water, and 3 mL of FRAP reagent, was incubated for 30 min at 37 °C in a water bath, and the absorbance was observed at 593 nm. The difference between sample absorbance and blank absorbance was calculated and used to calculate the FRAP value. FRAP values were expressed as mM Fe^2+^g^−1^ DW of the sample.

### 3.8. Sample Preparation for HPLC

For HPLC analysis, 2 mL of methanolic extract was evaporated using a Speed–Vac sample concentrator (model SPD 111 V, Thermo Savant, Waltham, MA, USA). The extracts were re-dissolved in HPLC-grade methanol, sonicated for 10 min with a sonicator (Sonar, Geneva, Switzerland), filtered through a nylon syringe filter (0.45 µm pore size, 25 mm diameter, Axiva Sichem Biotech, Delhi, India), and transferred in 300 µL sample vials.

#### High-Performance Liquid Chromatography (HPLC)

The HPLC system used for the separation of compounds was equipped with a PerkinElmer™ LC pump Series 200, a UV detector (N2910383, Perkin Elmer, Waltham, MA, USA) controlled with “PerkinElmer^®^’s TotalChrom^®^ Chromatography Data System (CDS) software”. Separation was accomplished on a Perkin Elmer Series 200 HPLC Peltier Column (5 µm) RP-18 column protected by a guard column of the same material. The HPLC analysis was performed using a solvent system as follows: Solvent A—0.0025% trifluoroacetic acid in water; Solvent B—80% acetonitrile (E. Merck, Mumbai, India) in Solvent A. The mobile phase consisted of Solvents (A) and (B). The step gradient solvent program was used as follows: 0–2 min: 85% A and 15% B; 2–5 min: 85% A and 15% B; 5–15 min: 80% A and 20% B; 15–20 min: 50% A and 50% B; 20–30 min: 40% A and 60% B; 30–35 min: 30% A and 70% B; 35–45 min: 20% A and 80% B; 45–48 min: 0% A and 100% B; 48–50 min: 0% A and 100% B; 50–55 min: 85% A and 15% B. Separation was performed at a flow rate of 1.0 mL/min, and chromatographic peaks were monitored at 254 nm [78,79]. The analyses of the samples were run in triplicate; identification is made by comparing the retention times and quantified by using standard curves for the peak area of the isoflavone standards.

### 3.9. Experimental Design and Statistical Analysis

The experiments were set up in a complete randomized block design. All the experiments were performed in triplicate, and results were averaged. Results were reported as the mean *±* standard deviation (SD) and analyzed by ANOVA followed by Duncan’s multiple range test (DMRT) at *p* ≤ 0.05 using SPSS statistical software version 16.0 (Statistical program for social sciences) program for windows (IBM, Armonk, NY, USA). To correlate the results obtained with different methods, a regression analysis was performed, and correlation coefficients were calculated. Microsoft office excels 2010 were used for data analysis.

## 4. Conclusions

This study investigated the changes in isoflavone content during sprouting, along with the effect of two precursors, Phe and *p*-CA, in relation to improved morphological characteristics, antioxidant activity, and isoflavonoid synthesis. The TPC, DPPH, and FRAP activities, as well as the morphological characteristics of chickpea sprouts, were significantly altered by different concentrations of *p*-CA and Phe. Compared to non-treated sprouts, *p*-CA at 250 mg L^−1^ and Phe at 500 mg L^−1^ were viable supplementations for increasing TPC, daidzin, and genistin contents.

Based on the findings of the present study, it can be concluded that extended sprouting up to the seventh day resulted in enhanced isoflavone content in control with no treatment, and both precursors have a non-significant effect on total isoflavone content, except at certain specific concentrations. The overall amount of isoflavones was similar between treated and untreated sprouts but higher than that in chickpea seeds. Therefore, incorporating chickpea sprouts with enhanced isoflavone content into the diet could offer a natural and convenient means of obtaining these health-promoting phytochemicals. Furthermore, the identification of unknown peaks in HPLC chromatograms may reveal new bioactive molecules that contribute to the radical scavenging activity of chickpea sprouts.

## Figures and Tables

**Table 1:** Length of hypocotyle (cm) and radical (cm) on the 3rd and 7th day in different concentrations of *p*-Coumaric acid.

Concentration (mg L^−1^)	Third Day			Seventh Day		
	Germination (%)	Length of Hypocotyle (cm)	Length of Radical (cm)	Germination (%)	Length of Hypocotyle (cm)	Length of Radical (cm)
Control	100	0.82 ± 0.60 ^a^	2.24 ± 0.35 ^a^	100	9.82 ± 2.37 ^a^	10.73 ± 3.34 ^a^
250	80	0.25 ± 0.21 ^b^	1.53 ± 0.51 ^b^	75	0.48 ± 0.30 ^b^	0.94 ± 0.19 ^b^
500	00	0.00 ± 0.00 ^c^	00 ± 00 ^c^	00	00 ± 00 ^c^	00 ± 00 ^c^
1000	00	0.00 ± 0.00 ^c^	00 ± 00 ^c^	00	00 ± 00 ^c^	00 ± 00 ^c^

Data analyzed the means ± standard deviation for statistical significance, and means in each column followed by the same letters are not significantly different, while different letters in each column are statistically different according to Duncan’s multiple range test (DMRT) at *p* ≤ 0.05.

**Table 2:** Length of hypocotyle (cm) and length of radical (cm) on the 3rd and 7th day in the different concentrations of L-phenylalanine.

Concentration (mg L^−1^)	Third Day		Seventh Day	
	Length of Hypocotyle (cm)	Length of Radical (cm)	Length of Hypocotyle (cm)	Length of Radical (cm)
Control	0.82 ± 0.60 ^ab^	2.24 ± 0.35 ^b^	9.82 ± 2.37 ^a^	10.73 ± 3.34 ^b^
250	0.84 ± 0.46 ^ab^	3.10 ± 0.84 ^a^	10.08 ± 4.06 ^a^	12.72 ± 3.96 ^a^
500	0.78 ± 0.54 ^b^	2.98 ± 1.43 ^a^	9.20 ± 2.60 ^a^	7.11 ± 4.03 ^c^
1000	0.87 ± 0.36 ^a^	2.35 ± 0.47 ^b^	9.28 ± 1.90 ^a^	12.11 ± 2.09 ^a^

Data analyzed the means ± standard deviation for statistical significance, and means in each column followed by the same letters are not significantly different, while different letters in each column are statistically different according to Duncan’s multiple range test (DMRT) at *p* ≤ 0.05.

**Table 3:** Effect of *p*-Coumaric acid (different concentration) on the total phenolic content and antioxidant capacity on different days on *C. arietinum* sprouts.

Day	Concentration (mg L^−1^)	TPC (mg GAE g^−1^ DW)	DPPH Redical Scavenging Activity (%)	FRAP (mM Fe^+2^ g^−1^ DW)
After soaking	Control	4.3 ± 0.53 ^d^	15.99 ± 0.54 ^d^	0.66 ± 0.61 ^d^
	250	7.9 ± 0.36 ^b^	27.96 ± 0.41 ^c^	1.31 ± 0.25 ^b^
	500	9.3 ± 0.91 ^a^	29.90 ± 0.84 ^b^	1.69 ± 0.53 ^a^
	1000	7.1 ± 0.56 ^c^	31.02 ± 0.63 ^a^	0.99 ± 0.39 ^c^
Third day	Control	5.6 ± 0.66 ^d^	37.27 ± 0.25 ^a^	0.27 ± 0.47 ^c^
	250	7.7 ± 0.42 ^b^	32.27 ± 0.69 ^b^	1.74 ± 0.42 ^a^
	500	7.2 ± 0.52 ^c^	19.47 ± 0.36 ^d^	1.39 ± 0.63 ^b^
	1000	8.2 ± 0.99 ^a^	26.98 ± 0.96 ^c^	1.76 ± 0.96 ^a^
Seventh day	Control	18.5 ± 0.36 ^a^	85.54 ± 0.43 ^a^	6.47 ± 0.52 ^b^
	250	17.6 ± 0.58 ^b^	52.45 ± 0.52 ^b^	7.34 ± 0.41 ^a^
	500	8.3 ± 0.42 ^c^	34.91 ± 0.94 ^c^	2.34 ± 0.54 ^c^
	1000	7.3 ± 0.91 ^d^	25.91 ± 0.69 ^d^	1.11 ± 0.11 ^d^

Data analyzed the means ± standard deviation for statistical significance, and means in each column followed by the same letters are not significantly different, while different letters in each column are statistically different according to Duncan’s multiple range test (DMRT) at *p* ≤ 0.05.

**Table 4:** Effect of L-phenylalanine (different concentration) on the total phenolic content and antioxidant capacity on different days on *C. arietinum* sprouts.

Day	Concentration (mg L^−1^)	TPC (mg GAE g^−1^ DW)	DPPH Redical Scavenging Activity (%)	FRAP (mM Fe^+2^ g^−1^ DW)
After soaking	Control	4.3 ± 0.53 ^d^	15.99 ± 0.54 ^b^	0.66 ± 0.61 ^d^
	250	8.1 ± 0.61 ^a^	12.38 ± 0.47 ^d^	2.17 ± 0.36 ^a^
	500	4.7 ± 0.29 ^b^	14.19 ± 0.35 ^c^	1.14 ± 0.35 ^b^
	1000	4.5 ± 0.72 ^c^	16.83 ± 0.27 ^a^	0.78 ± 0.66 ^c^
Third day	Control	5.6 ± 0.66 ^c^	37.27 ± 0.47 ^d^	0.27 ± 0.65 ^c^
	250	14.9 ± 0.22 ^b^	55.49 ± 0.92 ^c^	3.41 ± 0.99 ^a^
	500	14.7 ± 0.59 ^b^	60.64 ± 0.28 ^b^	3.07 ± 0.44 ^b^
	1000	15.9 ± 0.61 ^a^	74.83 ± 0.53 ^a^	3.46 ± 0.66 ^a^
Seventh day	Control	18.5 ± 0.36 ^c^	83.17 ± 0.35 ^a^	5.79 ± 0.25 ^a^
	250	24.3 ± 0.58 ^b^	80.95 ± 0.36 ^c^	3.78 ± 0.69 ^c^
	500	32.3 ± 0.42 ^a^	83.03 ± 0.58 ^a^	3.56 ± 0.49 ^d^
	1000	6.0 ± 0.91 ^d^	81.50 ± 0.85 ^b^	4.21 ± 0.53 ^b^

Data analyzed the means ± standard deviation for statistical significance, and means in each column followed by the same letters are not significantly different, while different letters in each column are statistically different according to Duncan’s multiple range test (DMRT) at *p* ≤ 0.05.

**Table 5:** Evaluation of isoflavonoid contents in *C. arietinum* sprouts in presence of two precursors by HPLC method (*p*-CA—coumaric acid; Phe—L-phenylalanine).

Sprouts	Daidzein (mg g^−1^)	Daidzin (mg g^−1^)	Genistein (mg g^−1^)	Genistin (mg g^−1^)	Biochain A (mg g^−1^)	Total Isoflavanoid (mg g^−1^)
Control, third day	0.076 ± 0.08 ^d^	0.29 ± 0.44 ^e^	1.05 ± 0.54 ^d^	0.411 ± 0.31 ^e^	0.219 ± 0.28 ^b^	2.046 ± 0.99 ^d^
*p*-CA 250 mg L^−1^, third day	*	0.73 ± 0.87 ^d^	*	1.057 ± 0.63 ^d^	*	1.787 ± 0.83 ^e^
Control, seventh day	0.507 ± 0.19 ^b^	5.8 ± 0.25 ^b^	2.3 ± 0.87 ^a^	3.945 ± 0.76 ^c^	0.267 ± 0.24 ^a^	12.819 ± 0.93 ^b^
Phe 250 mg L^−1^, seventh day	0.276 ± 0.11 ^c^	6.33 ± 0.27 ^a^	2.21 ± 0.59 ^b^	4.171 ± 0.88 ^b^	*	12.987 ± 0.86 ^a^
Phe 500 mg L^−1^, seventh day	0.534 ± 0.36 ^a^	2.75 ± 0.65 ^c^	1.99 ± 0.43 ^c^	6.842 ± 0.71 ^a^	*	12.116 ± 0.63 ^c^

* Content not determined. Data analyzed the means ± standard deviation for statistical significance, and means in each column followed by the same letters are not significantly different, while different letters in each column are statistically different according to Duncan’s multiple range test (DMRT) at *p* ≤ 0.05.

## Data Availability

The datasets used and/or analyzed during the current study are available from the corresponding author on reasonable request.

## Abbreviations

FW: fresh weight; DW: dry weight; *p*-CA: *p*-Coumaric acid; Phe: L-Phenylalanine; Zn: zinc; Se: selenium; Mg: magnesium; Ca: calcium; Cu: copper; K: potassium; P: phosphorus; PAL: phenylalanine ammonia-lyase; TPC: total phenolic content; GAE: gallic acid; DPPH; 2,2-Diphenyl-1-picrylhydrazyl; FRAP; ferric-reducing antioxidant potential; TPTZ: 2,4,6-Tris[2-pyridyl]-*s*-triazin; HPLC: high-performance liquid chromatography.
